# Diabetes and COVID19: a bidirectional relationship

**DOI:** 10.1038/s41430-021-00961-y

**Published:** 2021-06-23

**Authors:** Ranjit Unnikrishnan, Anoop Misra

**Affiliations:** 1 Department of Diabetology, Dr. Mohan’s Diabetes Specialities Centre & Madras Diabetes Research Foundation, Chennai, India; 2 Fortis-C-DOC Centre of Excellence for Diabetes, Metabolic Diseases and Endocrinology, New Delhi, India; 3 Diabetes Foundation (India), New Delhi, India; 4 National Diabetes Obesity and Cholesterol Foundation, New Delhi, India

**Keywords:** Diabetes complications, Diabetes complications

## Abstract

The advent and rapid spread of the coronavirus disease-2019 (COVID19) pandemic across the world has focused attention on the relationship of commonly occurring comorbidities such as diabetes on the course and outcomes of this infection. While diabetes does not seem to be associated with an increased risk of COVID19 infection per se, it has been clearly demonstrated that the presence of hyperglycemia of any degree predisposes to worse outcomes, such as more severe respiratory involvement, ICU admissions, need for mechanical ventilation and mortality. Further, COVID19 infection has been associated with the development of new-onset hyperglycemia and diabetes, and worsening of glycemic control in pre-existing diabetes, due to direct pancreatic damage by the virus, body’s stress response to infection (including cytokine storm) and use of diabetogenic drugs such as corticosteroids in the treatment of severe COVID19. In addition, public health measures taken to flatten the pandemic curve (such as lockdowns) can also adversely impact persons with diabetes by limiting their access to clinical care, healthy diet, and opportunities to exercise. Most antidiabetic medications can continue to be used in patients with mild COVID19 but switching over to insulin is preferred in severe disease.

## Introduction

Coronavirus disease 2019 (COVID19), caused by the novel severe acute respiratory syndrome-coronavirus 2 (SARS-CoV2) is the most significant pandemic to have affected humanity in the last 100 years. As of March 2021, more than 120 million documented infections have occurred worldwide, with more than 2.6 million deaths. As the COVID19 pandemic spreads, it appears likely that a significant proportion of the world’s population will be infected at some time or the other. Therefore, the interaction of COVID19 with other commonly occurring medical conditions needs to be studied so as to anticipate and thereby better manage the effects that they may have on one another.

Diabetes is one such comorbidity that affects more than 430 million people worldwide as of 2019 [1] and has the potential to unfavorably modify the natural history of COVID19. Conversely, COVID19 itself has been postulated to cause diabetes and to worsen glycemic control in pre-existing diabetes. Over the past year, a number of narrative, as well as systematic reviews analysing the link between diabetes and COVID19 infection, have been published [2–6], and their conclusions are summarized in Boxes 1 and 2. In this article, we attempt to build upon these earlier studies and critically analyse the bidirectional link between these two conditions, providing insights into the clinical implications of the relationship wherever applicable.

Box 1 Pre-existing diabetes and COVID19: risks

- No increased risk of COVID19 infection
- Increased risk of adverse infection outcomes (severe disease, ICU admission, need for assisted ventilation and mortality), even at levels of hyperglycemia lower than those suggestive of diabetes
- Increased risk of “long COVID” (tiredness, breathlessness, muscle and joint pain, inability to focus)

Box 2 COVID19 on diabetes: effects and use of antidiabetic medications

- Increased risk of development of new-onset hyperglycemia in infected individuals
- Worsening of glycemic control in pre-existing diabetes
- All classes of antidiabetic medications are suitable for use in mild cases of COVID19; insulin is to be preferred in severe cases

## Effects of hyperglycemia on COVID19

### Diabetes and risk of COVID

Individuals with diabetes do not seem to be at higher risk of being infected with SARS-CoV2, compared to the general population [2–4]. This is not entirely unexpected, as SARS-CoV2 generally affects the upper respiratory tract to begin with, and diabetes is in general not associated with a demonstrable increase in risk of upper respiratory tract infections (URTI) [5]. It is also possible that individuals with diabetes tend to follow non-pharmacological measures, such as mask wearing and social distancing more stringently than the general population, since they appreciate their higher risk of adverse outcomes of infection; this could have driven down infections in this population and masked a true increase in biological susceptibility.

#### Hyperglycemia and risk of adverse outcomes with COVID

In contrast to individuals without diabetes, COVID-19 infection in those with diabetes is associated with an increased risk of adverse outcomes such as more severe disease, pneumonia, ICU admissions, need for assisted ventilation and mortality. It is, however, not clear how much of this excess risk can be attributed to hyperglycemia, as people with diabetes (especially type 2 diabetes) have a higher frequency of other risk factors for severe COVID19 (such as advanced age, obesity, and cardiovascular disease) compared to those without diabetes.

#### A. Pre-existing diabetes and risk of adverse outcomes

It has been shown that among individuals with known diabetes, poor glycemic control preceding COVID19 infection (as measured by glycated hemoglobin levels) independently predicts adverse outcomes [6]. This reinforces the need for patients with all types of diabetes to maintain tight glycemic control during the pandemic period, a recommendation that might prove challenging to implement, given the difficulties in accessing chronic care, obtaining medications, and following diet and exercise guidelines during the pandemic and consequent lockdown [7]. The presence of diabetes mellitus was associated with a fourfold risk of adverse outcomes in a cohort of 339 patients from Wenzhou, China, even after adjusting for other potential confounders [8]. A clinic-registry based study from the United Kingdom showed that the presence of type 1 diabetes was associated with a 2.86-fold increased risk and that of type 2 diabetes with a 1.8-fold increased risk of dying from COVID19 compared to individuals without diabetes [9]. This study also showed that the presence of diabetes complications such as cardiovascular disease and nephropathy was independently associated with risk of COVID mortality. Preliminary studies indicate that diabetes might also be associated with increased risk of long-term sequelae of COVID19 such as tiredness, muscle and joint pain, breathlessness, and inability to focus (“long COVID”) [10].

#### B. Effect of mild degrees of hyperglycemia (“pre-diabetes”) on COVID outcomes

There have been several studies looking at the effect of hyperglycemia (not necessarily in the range diagnostic of diabetes) on adverse COVID outcomes. Even slightly raised blood glucose levels (in individuals not hitherto known to have diabetes) have been shown to be associated with poor outcomes in COVID19. Wang et al. [11] studied 605 patients from two hospitals in Wuhan, China and found that those with fasting blood glucose (FBG) of 6.1 To 6.9 mmol/l (mg/dL here) at admission had a nearly threefold increased odds of 28-day in-hospital complications, compared to those with FBG below 6.1 mmol/l. From Guangdong (China), Zhang et al. [12] showed that FBG at admission accurately predicted 30-day adverse outcomes (acute respiratory distress syndrome, multiple organ dysfunction, ICU admissions, septic shock, or death), irrespective of the presence or absence of pre-existing diabetes. Indeed, it has recently been suggested that blood glucose levels should be considered as a “vital sign” while evaluating hospitalized patients with COVID19 infection [13].

#### C. Effect of new-onset hyperglycemia on COVID outcomes

In a study from Wuhan, it was found that the risk of ICU admission was highest for patients with new-onset diabetes, followed by individuals with known diabetes and was lowest in those with new-onset hyperglycemia without diabetes [14]. However, Bode et al. [15] have shown that hyperglycemia without diabetes is associated with worse outcome in COVID19 compared to pre-existing diabetes. The poor outcomes associated with new-onset diabetes may reflect the fact that these hitherto undiagnosed and untreated patients would have more profound hyperglycemia and uncorrected metabolic risk factors compared to those with previously diagnosed diabetes [16]. The bulk of currently available evidence seems to suggest that new-onset hyperglycemia (irrespective of whether or not it meets the criteria for a diagnosis of diabetes) is associated with worse outcomes in COVID19 [17, 18].

#### Antidiabetic drug therapy in COVID19 infection

There have been no formal clinical trials comparing various antidiabetic agents in the context of COVID19 infection, so most of the available recommendations are based on expert opinion [19, 20]. In general, there is no contraindication for the use of any of the available antidiabetic agents in the setting of mild to moderate COVID19 infection, and the patient can continue with his/ her usual drug regimen, provided glycemic control is appropriate [21]. In severe COVID19 infection, it is always advisable to switch the patient to insulin therapy [21].

## Effect of COVID19 on hyperglycemia

### Hyperglycemia during COVID19 Infection

There is now sufficient evidence to conclude that COVID19 infection is a diabetogenic state. Reports from China during the initial days of the pandemic suggested that COVID19 infection is associated with new-onset diabetes, often presenting as diabetic ketoacidosis (DKA) or hyperosmolar hyperglycemic state (HHS) and requiring exceedingly large doses of insulin for control of hyperglycemia [16, 22, 23]. A global registry has now been established to study the link between COVID19 and new-onset diabetes [24]. Misra et al. [25] have recently attempted to broadly classify new-onset hyperglycemia during the COVID pandemic into those with documented COVID19 infection and those without. Among individuals with documented COVID19 infection, more severe hyperglycemia was found in those with severe infection, probably due to a combination of factors such as cytokine storm, corticosteroid use, and direct beta-cell damage.

**Fig. 1:**
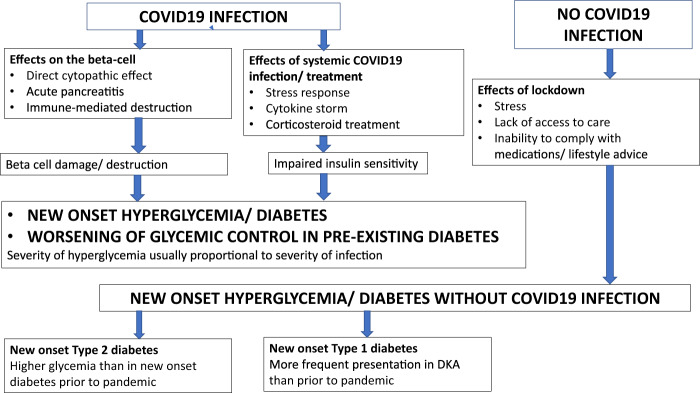
Hyperglycemic states during COVID pandemic.

The diabetogenic effect of COVID19 may manifest not only as new-onset diabetes, but also as worsening of pre-existing diabetes. The mechanisms for the development of hyperglycemia in COVID19 can be broadly enlisted as follows (Fig. 1).

#### Direct virus-mediated beta-cell damage

The angiotensin converting enzyme (ACE) receptor, which acts as the portal of entry for SARS-CoV2, has been identified not only on respiratory epithelial cells, but also in the kidney, gastrointestinal tract, and the pancreas. SARS-CoV2 has been shown to infect and replicate in cells of the human endocrine and exocrine pancreas [26]. Entry of SARS-CoV2 into the beta cells of the pancreas, with subsequent cell destruction, has been postulated to underlie the development of new-onset, insulin-requiring diabetes in some patients with COVID19. The fact that a similar phenomenon has been described with SARS-CoV1 adds credence to this hypothesis [27]. It is generally agreed that >90% of insulin-secreting cells need to be destroyed for this form of non-autoimmune diabetes to occur following a viral infection. In this respect, it is important to note that in contrast to SARS-Cov1, infection with SARS-CoV2 does seem to be associated with acute pancreatitis, with several cases having been reported in the literature [28, 29]. Clinical course of such kind of hyperglycemia remains to be researched.

#### Triggering of beta-cell autoimmunity by virus

In addition to direct cytopathic effects, viruses have been postulated to cause diabetes by triggering autoimmune attack against pancreatic beta-cell antigens. Indeed, this is one of the most prevalent hypotheses related to the etiopathogenesis of type 1 diabetes, with Coxsackie B virus, mumps virus, cytomegalovirus, rubella virus, and enteroviruses being the most commonly implicated agents. This theory holds that limited virus-mediated damage to beta cells releases hitherto sequestered antigens that lead to activation of autoreactive T-lymphocytes, culminating in an autoimmune response that ultimately destroys the remainder of the beta-cell mass, leading to insulin-dependent type 1 diabetes [30]. This process usually takes weeks to months and cannot explain the immediate onset of diabetes during the acute phase of COVID19 infection but may underlie disease development in some patients who develop diabetes in the weeks to months following recovery from infection. Research regarding this type of diabetes remains inadequate.

#### Diabetogenic effects of host responses to COVID19 infection

Host responses to COVID19 infection in the form of disorganized and exuberant immune response, can also lead to perturbations in glycemic status. As with any other acute infection, severe COVID19 is associated with non-specific activation of the immune system, with outpouring of counter-regulatory hormones and pro-inflammatory cytokines such as interleukin-6 (IL-6) and tumor necrosis factor (TNF) alpha, both of which are known to induce insulin resistance and hyperglycemia [31]. Sudden reduction in insulin sensitivity can precipitate diabetes in individuals with borderline beta-cell function and may even manifest as hyperglycemic crises in those with previously undiagnosed (and untreated) diabetes.

#### Iatrogenic hyperglycemia in COVID19

Following the publication of the interim findings of the Randomized Evaluation of Covid-19 Therapy (RECOVERY) trial, corticosteroids such as dexamethasone have become the mainstay of management of severe COVID19 infection [32]. While these drugs are highly effective in preventing clinical deterioration and death in COVID19 pneumonia, they are also known to be highly diabetogenic drugs, and hyperglycemia is virtually inevitable when they are used at the doses prescribed for this indication. In individuals with undiagnosed diabetes or pre-diabetes, profound hyperglycemia often leading to DKA or HHS can occur. While the presence of hyperglycemia cannot be considered a contraindication to corticosteroid use in a life-threatening situation such as COVID19 pneumonia, assessment of glycemic status is mandatory prior to using these drugs so that appropriate preventive/ therapeutic steps can be taken to minimize the magnitude of hyperglycemia.

### Hyperglycemia during COVID19 pandemic in individuals without COVID19 infection

In this context, it should not be forgotten that the COVID19 pandemic and the consequent policy responses such as lockdowns, have had profound effects on the lives of individuals with pre-existing diabetes. Analysis of a large French database showed that COVID-19-induced lockdowns were associated with far-reaching changes in dietary habits and physical activity levels, and these changes occurred in both favorable and unfavorable directions [33]. In many parts of the world, individuals with diabetes have faced difficulties in achieving requisite amounts of physical activity and accessing healthy foods during the pandemic [34]. Many of these patients would have put off their regular physician visits for fear of contracting the virus from the hospital or physician’s office. Deterioration in glycemic control is therefore not an unexpected consequence of the COVID19 pandemic, and predictions have been published as to the magnitude of this deterioration [35]. However, it is heartening to note that a few studies have actually shown improvement of glycemic control during the COVID pandemic [36, 37]. This may be due to increased awareness of the deleterious effects of diabetes particularly with reference to COVID, as well as patients having had more time to manage their diabetes in the “new normal” of working from home. There could, however, be a selection bias in these studies as only well-motivated patients would have ventured to seek clinic-based care during the lockdown.

Ghosh et al. [38] analyzed 282 patients with new-onset diabetes diagnosed during the COVID pandemic and compared them with 273 individuals newly diagnosed with diabetes immediately prior to the pandemic. They found that individuals with new-onset diabetes during the COVID pandemic had higher fasting and postprandial glucose levels and glycated hemoglobin compared to those diagnosed before the pandemic, even though the two groups did not differ significantly in any other respect including exposure to COVID (as measured by SARS-CoV2 antibody levels). Interestingly, based on data from multiple studies, Misra et al. [25] have stated that individuals without COVID19 infection who were diagnosed with either type 1 or type 2 diabetes during the pandemic, tended to present with more severe hyperglycemia than was the case prior to the pandemic (with a higher frequency of DKA in those presenting with type 1 diabetes). The worse metabolic profile of new-onset diabetes during the COVID pandemic is probably a reflection of increased stress, reduced physical activity and access to healthy foods and delayed diagnosis due to reluctance to visit physicians and hospitals during the pandemic and subsequent lockdown. It is likely that exacerbation in income inequalities following the lockdown will further worsen health outcomes, especially in countries like India where most healthcare is paid for “out-of-pocket” by the patient [39].

## Conclusions

The bidirectional relationship between COVID19 and hyperglycemia/diabetes presents a major challenge to healthcare systems as the pandemic spreads across the globe. It is essential that individuals with pre-existing diabetes get their blood glucose levels under control at the earliest so as to minimize adverse outcomes of COVID19, should they contract the infection. At the same time, physicians involved in the care of patients with COVID19 should be aware of the diabetogenic potential of this virus and look for new-onset hyperglycemia and diabetes in their patients, especially those treated with corticosteroids.
